# Associations between *DNAH1* gene polymorphisms and male infertility: A retrospective study

**DOI:** 10.1097/MD.0000000000013493

**Published:** 2018-12-10

**Authors:** Xiao Yang, Dongliang Zhu, Hongguo Zhang, Yuting Jiang, Xiaonan Hu, Dongfeng Geng, Ruixue Wang, Ruizhi Liu

**Affiliations:** a Center for Reproductive Medicine; b Center for Prenatal Diagnosis, First Hospital, Jilin University, Jilin, China.

**Keywords:** *DNAH1* gene, gene sequencing, genetic counseling, male infertility

## Abstract

Genetic abnormalities could account for 10% to 15% of male infertility cases, so increasing attention is being paid to gene mutations in this context. *DNAH1* gene polymorphisms are highly correlated with astheno-teratozoospermia, but limited information has been reported on pathogenic variations in *DNAH1* in the Chinese population. We explored 4 novel variations of the *DNAH1* gene in Chinese infertile patients. Mutation screening of the *DNAH1* gene was performed on 87 cases of asthenozoospermia with targeted high-throughput sequencing technology; another 200 nonobstructive azoospermia cases were further analyzed to investigate the prevalence of *DNAH1* variations. The effects of the variations on protein function were further assessed by bioinformatic prediction. For carriers of *DNAH1* variations, genetic counseling should be considered. Assisted reproductive technologies should be performed for these individuals and microsurgery should be considered for patients with azoospermia. *DNAH1* variations were identified in 6 of 287 patients. These included 8 heterozygous variations in exons and a splicing site. Among these, 4 variations (g.52400764G>C, g.52409336C>T, g.52430999_52431000del, g.52412624C>A) had already been registered in the 1000 Genomes and Exome Aggregation Consortium databases. The other 4 novel variations (g.52418050del, g.52404762T>G, g.52430536del, g.52412620del) were all predicted to be pathogenic by in silico analysis. The variations g.52418050del and g.52430999_52431000del were detected in 1 patient who was more severe than another patient with the variation g.52430999_52431000del. Physicians should be aware of genetic variants in male infertility patients and *DNAH1* mutations should be considered in patients with asthenospermia or azoospermia.

## 1 Introduction

Male infertility is a serious health problem that affects over 20 million men globally.^[1]^ It is caused by multiple factors, including genetic abnormalities, reactive oxygen species, immunological or endocrine diseases, varicocele, and infectious diseases.^[2–5]^ Genetic abnormalities could account for 10% to 15% of male infertility cases,^[6]^ so intensive efforts have been made to explore the relationship between genes and male fertility. However, only a few genes have been identified to correlate with defects in human sperm.^[7,8]^ The sperm flagellum is of fundamental importance to sperm motility and provides a forward driving force by its beating, so defects in the flagellum are closely related to male infertility. Moreover, mutations in several conserved dynein genes lead to defects of sperm flagellum of different levels of severity, being correlated with teratozoospermia or asthenozoospermia.^[9–11]^

*DNAH1* (MIM 603332) is one of these dynein genes, which encodes an inner dynein arm heavy chain. It is believed to strengthen the link between the outer doublet and the radial spokes, the latter of which is supposed to be responsible for localizing and stabilizing the central doublets.^[12]^ Pathogenic mutations in *DNAH1* could lead to the absence or dysfunction of DNAH1, causing severely disorganized assembly of the central doublets with axoneme.^[9]^ Such central pair defects correlated with a “9+0” axoneme structure were observed in patients with multiple morphological abnormalities of the sperm flagellum (MMAF).^[13]^ Numerous studies have reported that *DNAH1* mutations were identified in astheno-teratozoospermia patients with MMAF or female patients with primary ciliary dyskinesia (PCD).^[9,12,14,15]^ In addition, Ben Khelifa et al^[9]^ further proved that DNAH1 function is more critical in sperm flagellum than in cilium. *DNAH1* is thus an important candidate for a causative gene of male infertility. However, there have been only limited reports of *DNAH1* pathogenic variations in the Chinese.^[16–18]^

In the present study, we discovered several new *DNAH1* variations in Chinese male patients with infertility and made a prediction by bioinformatic analysis. This report extends the identified range of *DNAH1* variations associated with male infertility in the Chinese.

## 2 Patients and methods

### 2.1 Patients

A total of 87 idiopathic asthenozoospermia patients (World Health Organization semen motility grades of progressive motility [PR] + nonprogressive motility <40%; PR <32% in fresh ejaculate^[19]^) including several patients with combined oligo- and teratozoospermia were enrolled from May 2011 to April 2016. Exclusion criteria included medication, seminal infections, varicocele, systemic diseases, history of cryptorchidism or orchitis, and an abnormal karyotype. Moreover, in all subjects, the presence of antisperm autoantibodies was ruled out by using human antisperm antibody from the AsAb ELISA Kit (Beijing Beier Bioengineering Co., Ltd., Beijing, China). To fully investigate the prevalence of *DNAH1* variations in Chinese cases of male infertility, we further enrolled another 200 nonobstructive azoospermia patients diagnosed at our center from May 2011 to April 2016, applying the same exclusion criteria. The present study was approved by the Ethics Committee of the First Hospital of Jilin University. Written informed consent was provided by each participant before diagnosis.

### 2.2 Mutation screening

Samples of 5 to 10 mL of blood were collected from the patients into ethylenediaminetetraacetic acid anticoagulant tubes, followed by use of the BloodGen Midi Kit (Kangwei Century Biological Technology Co., Ltd., Beijing, China) for genomic DNA extraction.

Sequencing was carried out on all participants, using the Illumina MiSeq platform and an in-house targeted gene panel (Beijing Medriv Academy of Genetics and Reproduction, Beijing, China), which included the *DNAH1* gene. In accordance with previous references and OMIM databases, capture probes were also established based on the reported sequences of asthenozoospermia-associated genes. Fragments with a 3′/5′ linker and very small fragments with low quality were excluded using Cutadapt (https://pypi.python.org/pypi/cutadapt) and FastQC (https://www.bioinformatics.babraham.ac.uk/projects/fastqc/). The pre-processed clean reads were compared with the hg19 human reference sequence using BWA software (http://bio-bwa.sourceforge.net). Duplicated reads from library and PCR preparation were removed with Picard tools. For single nucleotide variants (SNVs) and indel variations in the pre-processed sequence, the Genome Analysis Tool Kit (https://www.broad institute.org/gatk) was further employed for analysis.

The calling quality was assessed using indices including align rate, duplication rate, rate of coverage ≥20× reading depth, and mean coverage, as follows: 100% align rate of over 95%, 100% duplication rate of <20%, rate of coverage ≥20× reading depth of between 99.9% and 92.0%, and mean coverage of the target region of >80×. Variations with population frequencies >1% were filtered using the 1000 Genomes (http://www.1000genomes.org/data), Exome Variation Server (http://evs.gs.washington.edu/EVS/), Exome Aggregation Consortium (http://exac.broad institute.org/), and dbSNP databases (http://www.ncbi.nlm.nih.gov/snp). With the exception of synonymous variations, both rare and novel variations were reviewed for further investigation. For the analysis of SNVs, SIFT (https://sift.jcvi.org/), PolyPhen-2 (http://genetics.bwh.harvard.edu/pph2/), and Mutation Taster2 (http://www.mutationtaster.org) algorithms were used to predict the no synonymous variations that would damage protein function. Mutation Taster2 was also used to assess frame shift variation and the harmfulness to splicing of mutations close to splicing sites was also predicted using Human Splicing Finder 3.1 (http://www.umd.be/HSF3/). The results were further validated with the use of Sanger sequencing (BGI, Shenzhen, China).

### 2.3 Analysis of DNAH1 variations reported

We searched the literature on DNAH1 variations in infertile men using PubMed, and then analyzed the relationship between DNAH1 variations and male infertility.

## 3 Results

A total of 6 of 287 (2.09%) patients were found to have *DNAH1* variations. The general information and semen parameters of the patients are presented in Table 1.

**Table 1:**
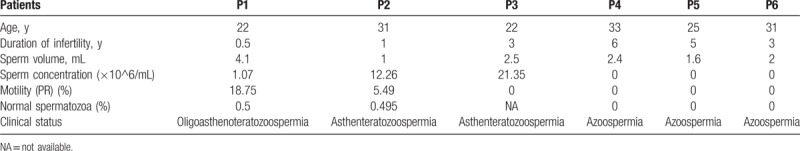
General information and semen parameters of infertile patients with *DNAH1* gene mutations.

Targeted gene capturing and high-throughput sequencing were performed to detect the potentially deleterious variations in 87 idiopathic astheno-teratozoospermia patients. To study the genetic pathogeny of male infertility, we chose 52 genes altogether (Table 2) reported to be related to this condition including *DNAH1* as candidate genes by reviewing the literature. According to a previous study on *DNAH1*, the gene contains 78 exons and encodes a dynein protein comprising 4265 residues.

**Table 2:**
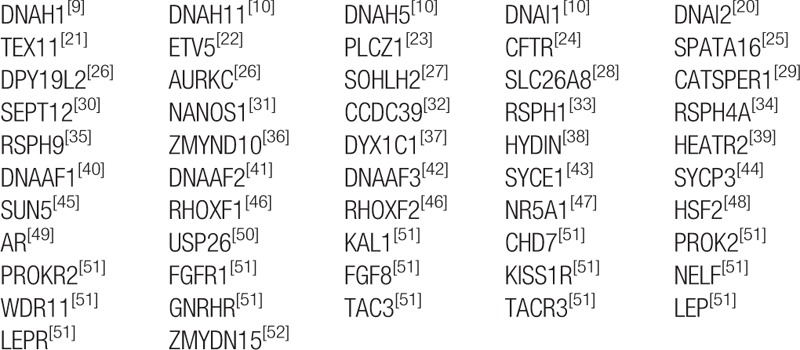
Lists of 52 genes tested in the study.

**Figure 1:**
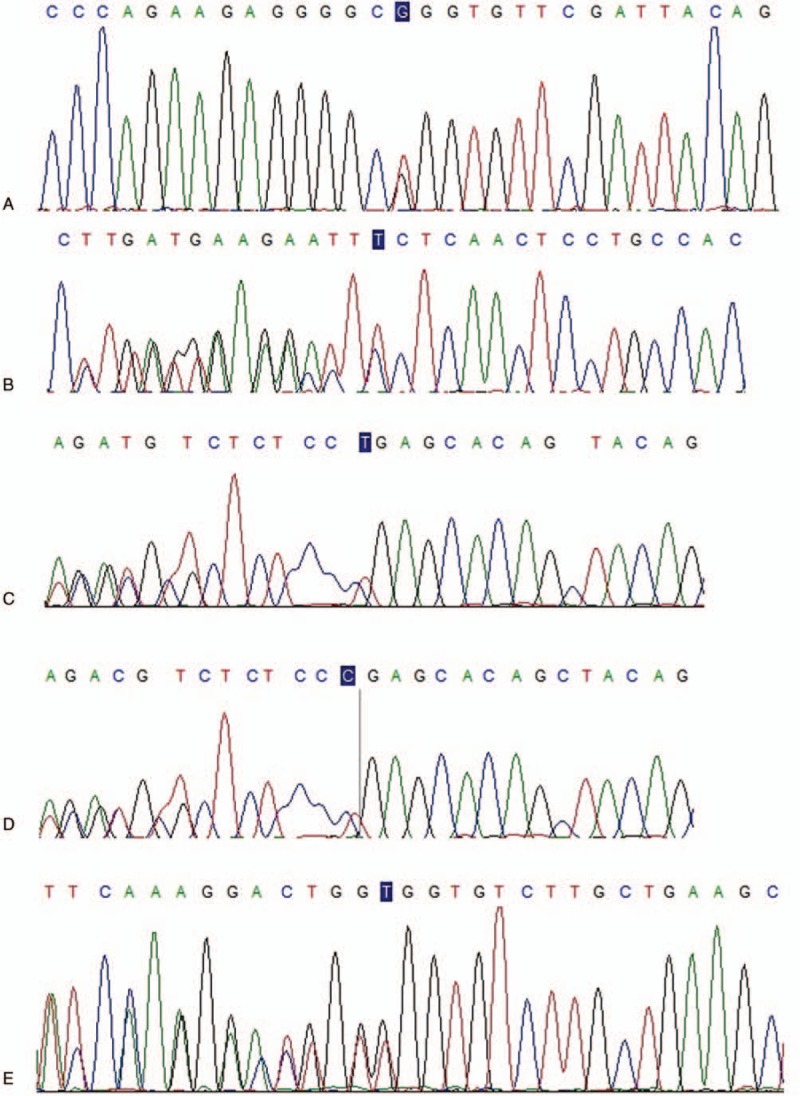
Sequencing maps of DNAH1 gene variations. A: g.52404762T>G (P4); B: g.52430536del (P5); C: g.52430999_52431000del (P2); D: g.52430999_52431000del (P3); E: g.52418050del (P3).

**Figure 2:**
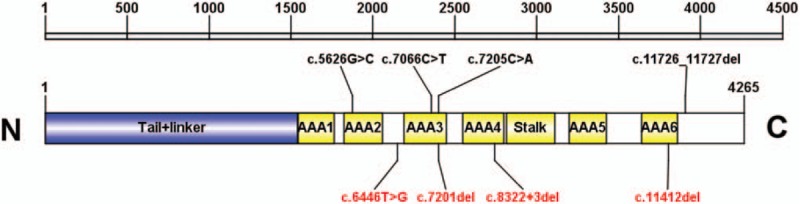
Location of variations in DNAH1 domain structures. The 6 AAA-domains (AAA1–6) together with the coiled-coil stalk constitute a conserved dynein motor domain. Six variations (g.52400764G>C, g.52409336C>T, g.52412620del, g.52412624C>A, g.52418050del, g.52430536del) were respectively located at 6 AAA-domains; and 4 novel identified variations are listed in red.

We identified four *DNAH1* heterozygous variations in 3 cases (P1–P3, Table 3; Fig. 1). No harmful variations of other candidate genes were found in these patients. Three (g.52400764G>C, g.52409336C>T, g.52430999_52431000del) of these 4 variations had already been registered in the 1000 Genomes (http://browser.1000genomes.org) and ExAC databases (http://exac.broadinstitute.org). The other splicing variation g.52418050del located in a splice site of intron 52 was identified in P3; this variation was predicted by Human Splicing Finder 3.1 (http://www.umd.be/HSF3/) to affect splicing. We further identified another 4 variations in 3 nonobstructive azoospermia patients (P4–P6, Table 3; Fig. 1). Three of these (g.52404762T>G, g.52430536del, g.52412620del) had never previously been reported. More details of these are presented in Table 3. The locations of these variations are presented in Fig. 2. Overall, 8 *DNAH1* variations were detected in the infertile patients; among them, 6 variations (g.52400764G>C, g.52409336C>T, g.52412620del, g.52412624C>A, g.52418050del, g.52430536del) were respectively located at 6 AAA-domains, which together with a coiled-coil stalk constitute a conserved dynein motor domain. Given their potential pathogenicity as predicted by bioinformatics’ analyses, we focused more on these variations and analyzed them further.

**Table 3:**
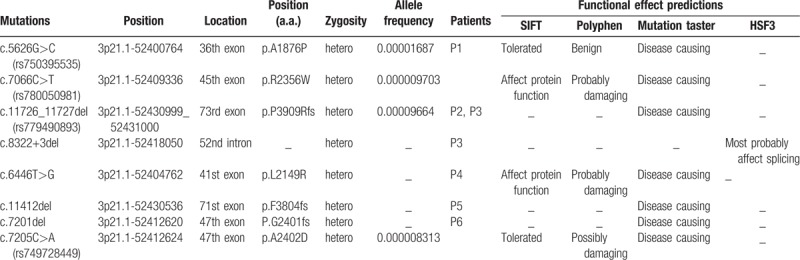
Bioinformatic analysis of *DNAH1* variations identified by whole-genome sequencing.

## 4 Discussion

Sperm flagellum is highly complex, having a series of proteins responsible for its assembly, composition, and function. Defects in sperm flagellum caused by exogenous and endogenous factors including genetic ones can lower sperm motility.^[53,54]^ However, it is difficult to identify the association between altered sperm motility and gene mutations. *DNAH1*, one of several dynein members highly correlated with sperm dysmotility, has been confirmed to be an important candidate gene for male infertility. Neesen et al^[8]^ first suggested that patients with mutations in *DNAH1* could suffer from asthenozoospermia. Subsequently, from 2014 to 2017, a range of studies revealed the pathogenicity of several specific mutations in *DNAH1*.^[12,14,17,18,55]^ Among 3 convincing studies about the infertile population in China and *DNAH1* mutations, those by Sha et al^[17]^ and Wang et al^[18]^ reported a specific correlation of a total of 16 asthenozoospermia patients with MMAF and *DNAH1* mutations, while the study by Yang et al^[16]^ only sequenced 4 exons of the *DNAH1* gene. In these former studies, a total of 11 of 16 (68.75%) patients carried *DNAH1* heterozygous mutations. Among Chinese infertile patients, heterozygous mutations in *DNAH1* are thus potential causes of the infertility.

Sha et al^[17]^ demonstrated that the heterozygous variations g.52430999_52431000del and g.52409336C>T are associated with asthenozoospermia in patients with MMAF. In our study, we found 4 potential heterozygous variations in 3 astheno-teratozoospermia patients. The compound heterozygous variationsg.52409336C>T and g.52400764G>C were detected in patient P1. The variation g.52400764G>C in exon 36 may result in p.A1876P, which was predicted to be benign. Given the pathogenicity of the variation g.52409336C>T, in patient P1 diagnosed with oligoastheno-teratozoospermia, this condition might partially have resulted from the compound heterozygous variation. In addition, the variation g.52430999_52431000del was identified in patients P2 and P3. Moreover, g.52418050del (c.8322+3del) is a novel pathogenic variation found in patient P3, which was predicted to affect splicing. In terms of sperm progressive motility, patient P3 showed a more severe phenotype than P2 (0 vs 5.49% motile sperm, Table 1), which suggests that the compound heterozygous variations (g.52430999_52431000del and g.52418050del) have more severe effects.

A possible link between azoospermia and variations in the dynein genes, such as *DNAH5*, has been reported.^[56,57]^ Considering this, we further investigated the prevalence of *DNAH1* variations in Chinese infertile patients, including another 200 azoospermia patients, at our center. Overall, 2.09% of patients were found to carry *DNAH1* variations, and another 4 pathogenic variations identified by bioinformatics’ analyses were detected in 3 azoospermia patients (P4–P6, Table 3). Among these, the *DNAH1* deletions g.52430536del in exon 71 and g.52412620del in exon 47 cause frame shifts, p.F3804fs and p.G2401fs, respectively. The other 2 variations were missense mutations, c.6446T>G (g.52404762T>G) and c.7205C>A (g.52412624C>A), which may have detrimental effects on protein function. However, with the development of assisted reproduction technologies, these patients may be able to have their own children. Unfortunately, owing to the role of these mutation sites, their inheritance by the next generation would be associated with a high probability of infertility.

## 5 Conclusion

In the present study, 4 novel variations (g.52418050del, g.52430536del, g.52412620del, g.52404762T>G) are reported in Chinese male infertility patients. Physicians should be aware of the possibility of male infertility patients having genetic variants causative of their condition, and *DNAH1* mutations in particular should be considered in patients with asthenospermia or azoospermia.

## Author contributions

**Conceptualization**: Xiao Yang, Dongliang Zhu.

**Data curation**: Hongguo Zhang.

**Funding acquisition:** Hongguo Zhang, Ruizhi Liu.

**Investigation:** Xiao Yang, Yuting Jiang, Ruixue Wang.

**Methodology**: Yuting Jiang, Xiaonan Hu, Dongliang Zhu, Dongfeng Geng

**Resources**: Dongfeng Geng, Ruixue Wang

**Writing – original draft**: Xiao Yang

**Writing – review & editing**: Ruizhi Liu
